# Development of a Prognostic Five-Gene Signature for Diffuse Lower-Grade Glioma Patients

**DOI:** 10.3389/fneur.2021.633390

**Published:** 2021-07-06

**Authors:** Qiang Zhang, Wenhao Liu, Shun-Bin Luo, Fu-Chen Xie, Xiao-Jun Liu, Ren-Ai Xu, Lixi Chen, Zhilin Su

**Affiliations:** ^1^Department of Clinical Laboratory, The People's Hospital of Lishui, Lishui, China; ^2^Guangdong-Hong Kong-Macao Greater Bay Area (GBA) Research Innovation Institute for Nanotechnology, Guangzhou, China; ^3^Department of Clinical Pharmacy, The People's Hospital of Lishui, Lishui, China; ^4^Department of Urinary Surgery, The People's Hospital of Lishui, Lishui, China; ^5^Pathology Department, The People's Hospital of Lishui, Lishui, China; ^6^The First Affiliated Hospital of Wenzhou Medical University, Wenzhou, China; ^7^Department of Gynecology in Xiahe Branch, Xiamen University Affiliated Zhongshan Hospital, Xiamen, China; ^8^Department of Laboratory Medicine, The First Affiliated Hospital of Xiamen University, Xiamen, China

**Keywords:** lower-grade glioma, signature, prognostic biomarker, survival, gene expression

## Abstract

**Background:** Diffuse lower-grade gliomas (LGGs) are infiltrative and heterogeneous neoplasms. Gene signature including multiple protein-coding genes (PCGs) is widely used as a tumor marker. This study aimed to construct a multi-PCG signature to predict survival for LGG patients.

**Methods:** LGG data including PCG expression profiles and clinical information were downloaded from The Cancer Genome Atlas (TCGA) and the Chinese Glioma Genome Atlas (CGGA). Survival analysis, receiver operating characteristic (ROC) analysis, and random survival forest algorithm (RSFVH) were used to identify the prognostic PCG signature.

**Results:** From the training (*n* = 524) and test (*n* = 431) datasets, a five-PCG signature which can classify LGG patients into low- or high-risk group with a significantly different overall survival (log rank *P* < 0.001) was screened out and validated. In terms of prognosis predictive performance, the five-PCG signature is stronger than other clinical variables and IDH mutation status. Moreover, the five-PCG signature could further divide radiotherapy patients into two different risk groups. GO and KEGG analysis found that PCGs in the prognostic five-PCG signature were mainly enriched in cell cycle, apoptosis, DNA replication pathways.

**Conclusions:** The new five-PCG signature is a reliable prognostic marker for LGG patients and has a good prospect in clinical application.

## Introduction

Glioma is the most common primary CNS tumor and was classified into grades I–IV according to histopathological characteristics. Glioblastoma (WHO grade IV glioma) accounts for 70–75% of all diagnosed diffuse gliomas, with a median overall survival of 14–17 months (1). Diffuse low-grade (WHO grade II) and intermediate-grade (WHO grade III) gliomas are considered lower-grade gliomas (LGGs), and their clinical behavior is highly variable, with a prognosis of 1–15 years (2). Overall, the prognosis of glioma patients is not satisfactory. For LGGs, the great prognostic variance among patients subjected to the same therapeutic regimen is the highlighted clinical problem. Thus, identification of patients with bad survival is very important for instructing subsequent treatment.

Glioma is a fatal tumor that derives from glial cell and grows in the central nervous system, including diffuse gliomas and nondiffuse gliomas (3). Diffuse gliomas are the most frequently occurring intracranial malignant tumors, encompassing various histologic types (astrocytic or oligodendroglial) and malignancy grades [World Health Organization (WHO) grades II, III, and IV] tumors. Astrocytomas and oligodendrogliomas in the low grade (WHO II) and intermediate grade (WHO III) are incorporated into diffuse lower-grade glioma (LGG) and perform better than IV grade glioblastoma in both malignancy and prognosis. However, it is difficult to predict the clinical outcome of LGG patients because LGG are a highly heterogeneous group of tumors. Firstly, there is a difference in the speed of tumor progression within LGG. Some are relatively inert, while others quickly progress to high-grade glioma or glioblastoma. Secondly, therapeutic sensitivity varies in LGG patients. Some people have effective treatments, while others have poor treatment results. Finally, LGG patients differ greatly in the prognosis, ranging from 1 to 15 years (2). Due to the limitations of histologic classification of LGG, finding molecular markers that can accurately predict prognosis and treatment response has become an urgent task (4).

In recent years, significant progress has been made in the study of molecular pathology of gliomas, and a series of molecular markers have been discovered that are helpful for clinical diagnosis, prognostic judgment, and treatment guidance, such as IDH1/2 gene mutation, chromosome 1p/19q co-deletion, and MGMT promoter methylation (5). Especially, the revised 2016 WHO classification of CNS tumors made fundamental changes and classified diffuse gliomas based on IDH mutation and 1p/19q co-deletion status. This innovative measure highlights the important role of novel and reliable gene biomarkers in the diagnosis and prognosis of gliomas.

With the development of next-generation sequencing technology, a large amount of high-throughput sequencing data and a variety of bioinformatics methods have prompted researchers to further understand tumorigenesis and find prognostic markers. Hu et al. (6) selected a prognostic 35-gene signature from 374 glioma patients carrying the 1p/19q co-deletion. Wu constructed a six-gene signature that could classify IDH-mutant GBM patients into high or low risk of poor outcome using 33 samples from the Chinese Glioma Genome Atlas RNA-sequencing data and 21 cases from Chinese Glioma Genome Atlas microarray data (7). Deng found a four-gene immune prognostic signature for predicting prognosis in LGGs through analyzing 511 LGG samples from the TCGA database and 172 LGG samples from the CGGA dataset (8). Therefore, gene signature has become the research focus of glioma prognostic markers.

In the present study, the protein-coding gene (PCG) expression data from a total of 955 LGG patients were collected from the Cancer Genome Atlas (TCGA) database and the Chinese Glioma Genome Atlas (CGGA). We aimed to mine the large queue of gene expression data and clinical information to identify a prognostic PCG signature and explore its significance of treatment guidance.

## Materials and Methods

### Data Collection of Diffuse LGG Patients

The clinical information and mRNA expression data of LGG patients were obtained from the TCGA database (http://cancergenome.nih.gov/; https://xenabrowser.net/datapages/). Another independent dataset used as validation or test dataset was downloaded from the CGGA database (http://www.cgga.org.cn/). LGG cases with clinical survival information including survival status and survival time were selected for building the prognostic model. Clinical details of LGG patients in the training and test datasets are shown in Supplementary Table 1. Genes with missing expression values in >20% samples were removed in subsequent analysis (9).

### The Process of Developing the Prognostic Signatures in the Training Dataset

Using Kaplan–Meier (KM) and receiver operating characteristic (ROC) analysis, we identified the PCGs significantly associated with patients' OS with AUC > 0.6 from the TCGA group. Then we reduced the number of the PCGs by the random survival forest algorithm (RSFVH). Further, prognostic models were constructed as follows:

$$Riskscore=\sum_{i\;=1}^{N}\left(Expression_{i}\times coefficient_{i}\right)$$

where *N* is the number of PCG, Expression is the PCG expression value, and coefficient is the PCG expression in Cox regression analysis. The final prognostic PCG signature was screened out with the largest AUC value in all the constructed models (10).

### Statistical and Bioinformatics Analysis

Kaplan–Meier analysis was used to assess the two survival risk groups separated by the median risk score. Cox regression analysis was performed to explore the independence of the signature. ROC and TimeROC were used to analyze survival prediction performance. Function prediction of prognostic PCGs was analyzed by clusterProfiler (11). R program (www.r-project.org) with R packages including pROC, TimeROC, randomForestSRC, and survival was used to perform the above analyses.

## Results

### The Process of Developing the Prognostic Signatures in the Training Dataset

All 955 patients diagnosed with LGG were collected from the TCGA (*n* = 524) and CGGA (*n* = 431) datasets, and a total of 16,246 expressed PCGs were identified. From Table 1, we found that the median age of the enrolled patients was 40 years (11–87 years) and that there were more male patients than female patients, indicating that LGG is more likely to occur in adult males. When focusing on the survival status and survival time of these patients, we found that more than one-third of patients (326 of 955) had died and the median survival time was only 2.11 years (0.2–14.15 years). In addition, we also obtained IDH mutation status, 1p19q co-deletion, radiotherapy, and chemotherapy information for further analysis.

**Table 1:** Relationship of the five-gene signature with features in the two groups with LGG.

**Feature**	**Training set**		***P***	**Test set**		***P***
	**Low^**#**^**	**High^**#**^**		**Low^**#**^**	**High^**#**^**	
**Age (years)**			0.02			0.99
≤ 40	144	117		111	110	
>40	118	145		105	105	
**Gender**			0.99			0.88
Female	119	118		98	95	
Male	143	144		118	120	
**Grade**			<0.001			<0.001
G2	169	88		111	69	
G3	92	174		105	146	
Unknown	1	0		0	0	
**IDH mutation status**			<0.001			<0.001
Mutant	69	22		183	114	
Wild type	9	25		13	83	
Unknown	184	215		20	18	
**Radiotherapy**			<0.001			0.31
No	119	54		47	39	
Yes	113	171		157	157	
Unknown	30	37		12	19	
**Chemotherapy**						0.04
No				74	50	
Yes				124	141	
Unknown				18	24	
**1p19q co-deletion status**						<0.001
Co-deletion				99	29	
Non-co-deletion				98	167	
Unknown				19	19	

# *The median risk score was used to classify patients into low- and high risk groups*.

**Figure 1:**
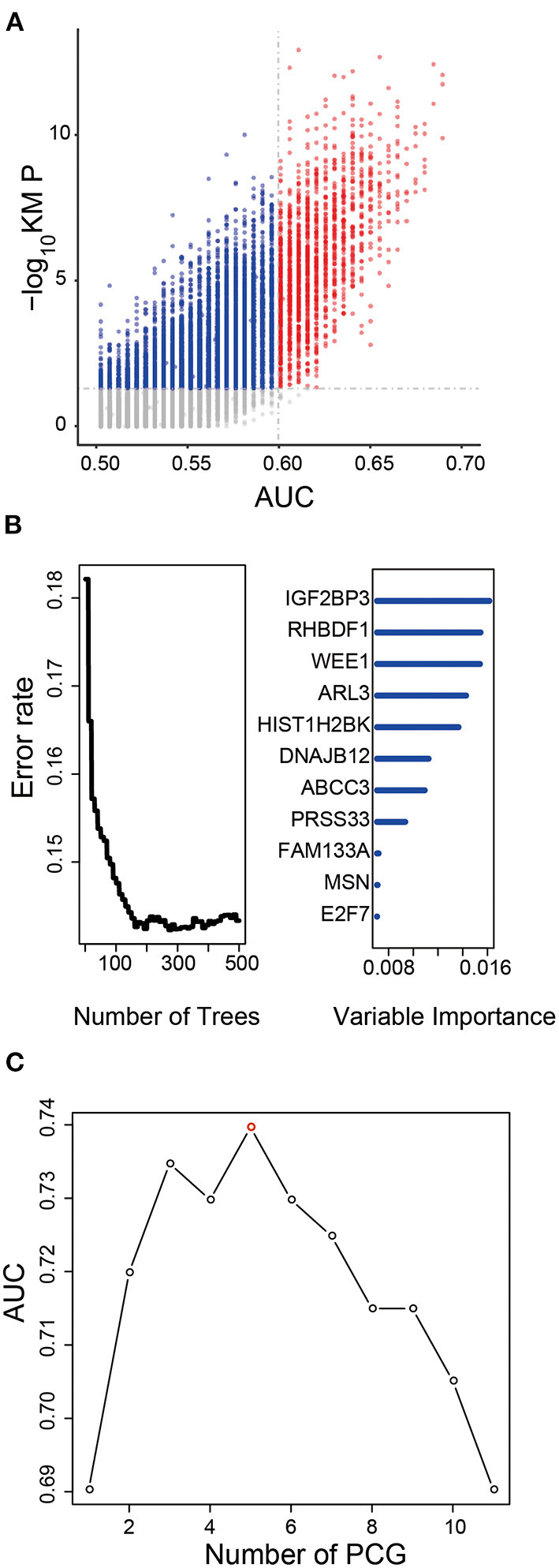
Development of the prognostic signature in the training dataset. **(A)** The survival-associated PCGs in Kaplan–Meier analysis were displayed as red dots in the scatter diagram. **(B)** Random forest supervised classification algorithm reduced the prognosis-associated PCGs to 11 PCGs. **(C)** The prognostic five-PCG signature was selected because its AUC was the largest (AUC = 0.739) among the 2^11^−1 = 2,047 signatures.

After Kaplan–Meier and ROC analysis in the TCGA dataset, a total of 1,702 PCGs were discovered (red dots in Figure 1A), which were significantly associated with OS and had a good ability to predict survival (KM *P* < 0.05 and AUC > 0.6, Supplementary Table 2). Further, we screened out 11 prognostic PCGs by RSFVH analysis based on importance scores (Figure 1B). Then, we brought the prognostic PCGs into the risk prediction model and got 2^11^-1 = 2,047 possible signatures in the training dataset. ROC analyses were performed in all the 2,047 signatures to find out the signature with the strongest predictive ability (Supplementary Table 3). The final signature including five PCGs (ABCC3, SMC4, EMP3, WEE1, and HIST1H2BK) related to LGG prognosis significantly (Supplementary Figure 1A) was screened out with the maximum AUC (AUCsignature = 0.739; Figure 1C). The selected risk model is as follows: risk score = (0.28 × expression value of ABCC3) + (0.66 × expression value of SMC4) + (0.44 × expression value of EMP3) + (0.61 × expression value of WEE1) + (0.45 × expression value of HIST1H2BK). In addition, the survival curves with univariable Cox hazard ratio for each gene in the signature in the CGGA group are also shown in Supplementary Figure 1B. The five genes, significantly associated with LGG prognosis, were also observed in the CGGA dataset. The result suggested that the five genes were reliable prognostic biomarkers for patients with LGG.

### The Performance of PCG Signature in Predicting LGG Patient Survival

**Figure 2:**
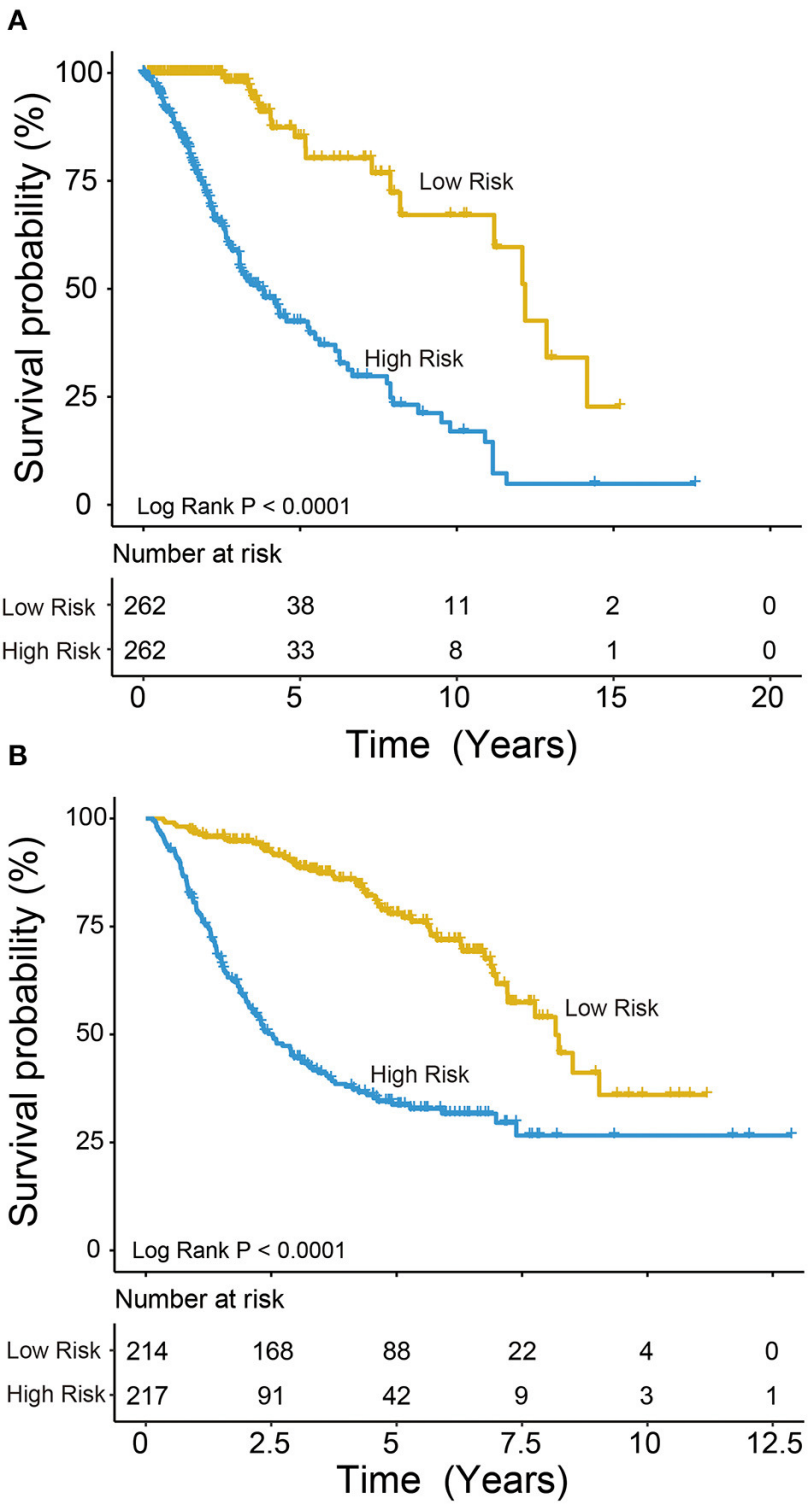
Kaplan–Meier plots indicated that LGG patients could be classified into high- and low-risk groups according to the five-gene signature in the training **(A)** and test **(B)** datasets.

**Figure 3:**
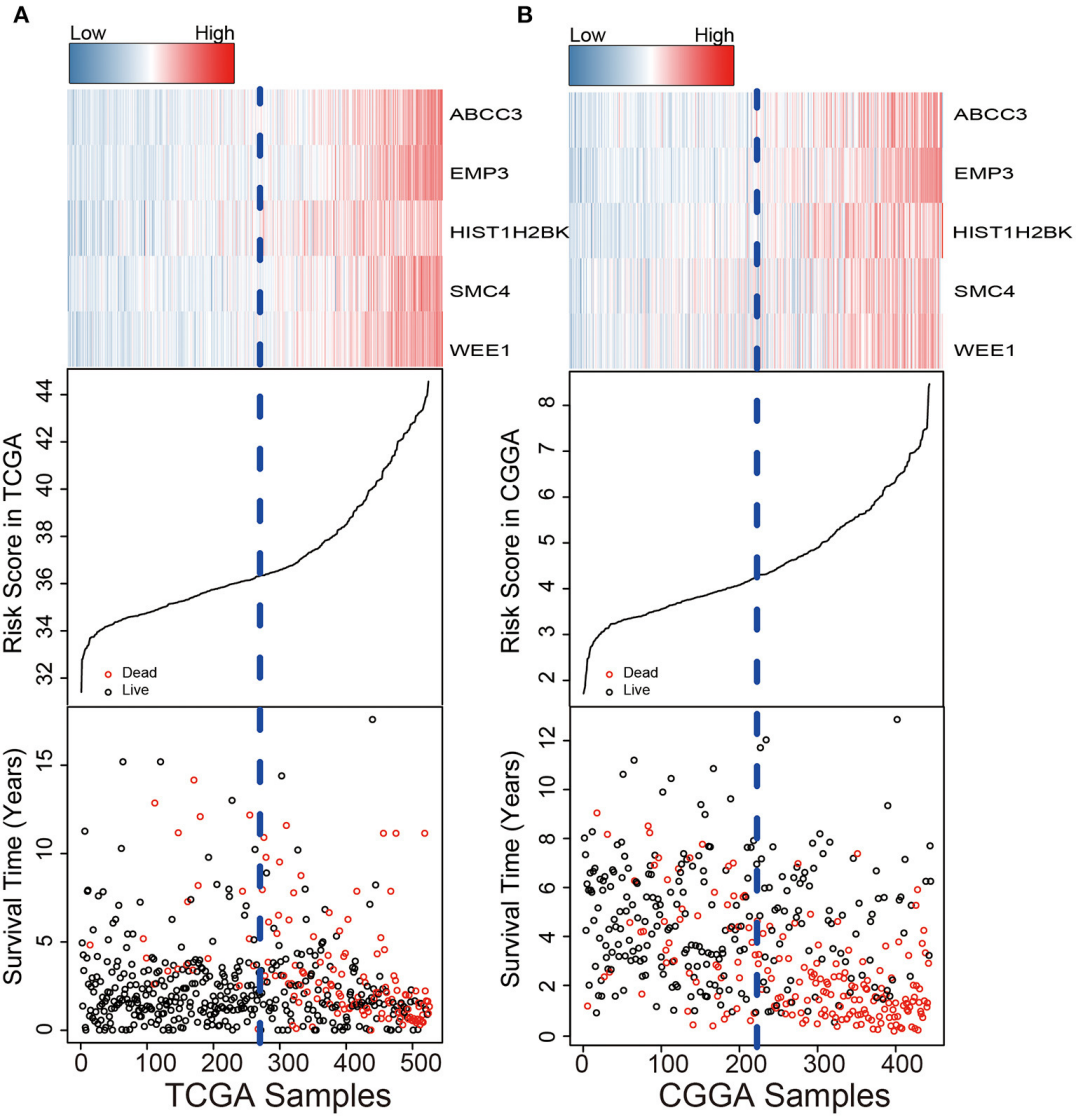
Risk score distribution, survival status, and PCG expression patterns for LGG patients in the training **(A)** and test **(B)** datasets.

We used the risk model to calculate the risk scores for each patient. The median risk score was used to divide patients in the training dataset into either the high-risk (*n* = 262) or low-risk group (*n* = 262). The Kaplan–Meier analysis results showed that patients in the low-risk group lived longer than patients in the high-risk group (median survival time: 12.18 years vs. 3.84 years, *P* < 0.001; Figure 2A). Then, we tested the prognostic value of the PCG signature in another large independent LGG dataset (CGGA, *n* = 431). After the median risk score in CGGA-separated patients into high- or low-risk group, Kaplan–Meier analysis found that the 5-year survival of patients with high risk scores was lower than that of patients with low risk scores (5-year survival: 34.16 vs. 77.05%, log-rank test *P* < 0.001; Figure 2B). We showed the relationship of PCG expression, risk score, and survival information in Figure 3. With the increase of gene expression value, risk scores and death toll increased in the training (Figure 3A) and test datasets (Figure 3B).

### The Five-PCG Signature Is an Independent Predictive Factor

In the two LGG groups (*n* = 524/431), we found that the signature was related with clinical variables such as IDH mutation status and Grade by chi-square test (*P* < 0.001; Table 1). In addition, we found that the 1p19q co-deletion status could predict the patients with LGG significantly (Supplementary Figure 2) and the signature was also associated with 1p19q co-deletion status based on the CGGA dataset (*P* < 0.001; Table 1). Then, we further performed univariate and multivariable Cox regression analyses to test the predictive independence of the signature. Multivariable Cox regression results verified that the signature was an independent predictive factor and could independently predict patients' clinical outcome in training or test datasets (high- vs. low-risk, HR training = 1.70, 95% CI 1.31–2.21, *P* < 0.001, *n* = 524; HR test = 3.01, 95% CI: 2.12–4.27, *P* < 0.001, *n* = 431; Table 2).

**Table 2:** Univariable and multivariable Cox regression of the signature with patient survival in two LGG datasets.

**Variables**		**Univariable**				**Multivariable**			
		**HR**	**95% CI of HR**		***P***	**HR**	**95% CI of HR**		***P***
			**Lower**	**Upper**			**Lower**	**Upper**	
**TCGA set**									
Age	>40 vs. ≤ 40	2.82	1.96	4.04	<0.001	1.99	0.52	7.60	0.32
Gender	Male vs. female	1.14	0.81	1.60	0.45	2.00	0.66	6.09	0.22
IDH status	Wild type vs. mutant	5.53	2.07	14.82	<0.001	0.94	0.22	4.07	0.94
LGG Grade	G3 vs. G2	3.31	2.28	4.79	<0.001	0.79	0.22	2.81	0.72
Signature	High risk vs. low risk	6.86	4.26	11.04	<0.001	1.70	1.31	2.21	<0.001
**CGGA set**									
Age	>40 vs. ≤ 40	1.19	0.89	1.58	0.24	1.10	0.82	1.48	0.54
Gender	Male vs. female	1.00	0.75	1.34	0.98	1.14	0.85	1.54	0.38
IDH status	Wild type vs. mutant	2.24	1.64	3.07	<0.001	1.48	1.06	2.07	0.02
Grade	G3 vs. G2	2.62	1.89	3.64	<0.001	2.58	1.81	3.66	<0.001
Signature	High risk vs. low risk	3.68	2.69	5.03	<0.001	3.01	2.12	4.27	<0.001

### Predictive Performance Comparison Between the Five-PCG Signature With Other Clinical Variables

**Figure 4:**
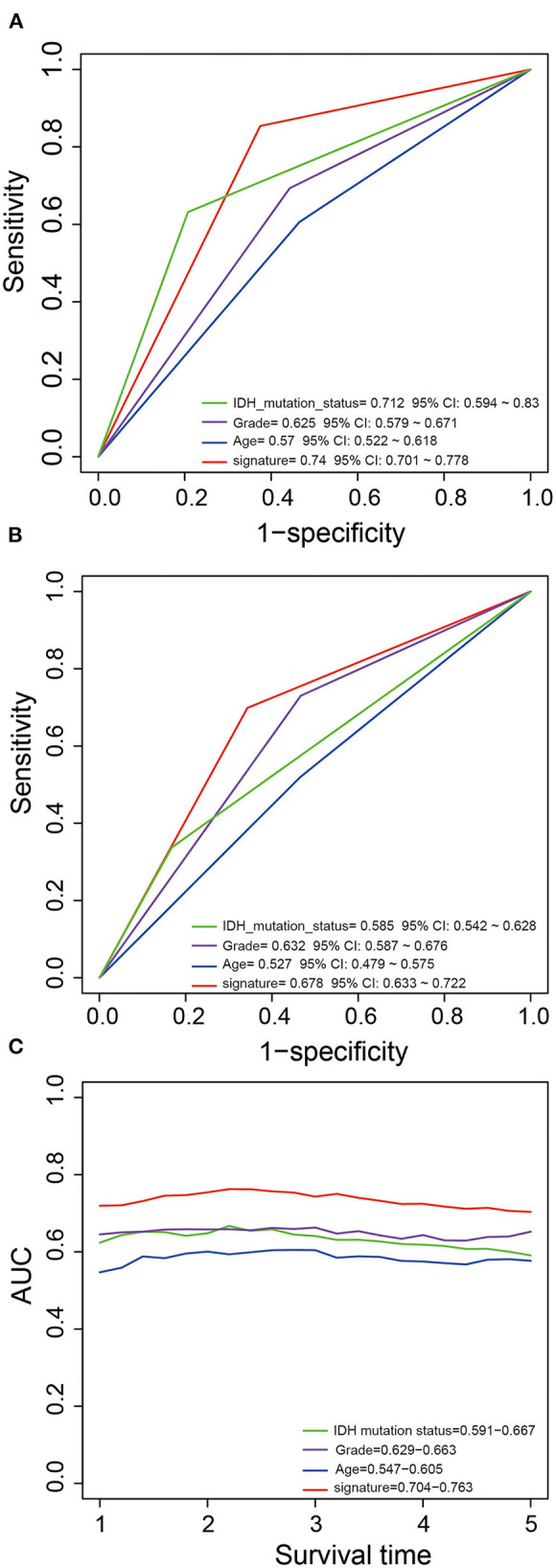
Comparison of the survival predictive power of the signature with grade, age, and IDH mutation by ROC in the training **(A)** and test **(B)** sets. TimeROC analysis of survival predictive power for the signature, grade, age, and IDH mutation **(C)**.

We performed ROC analysis to compare the predictive performance of the five-PCG signature with other clinical variables including IDH mutation status, age, and grade. Figures 4A,B shows that the PCG signature outperformed the above clinical variables in both the training and test sets (AUCsignature 0.739/0.678 vs. AUCIDH 0.712/0.585; AUCgrade 0.625/0.632; AUCage 0.57/0.527). Further, TimeROC analysis found that the AUC values of the signature from 1 to 5 years were greater than that of IDH mutation status, grade, or age, indicating that the PCG signature had better survival prediction when integrating the TCGA and CGGA datasets (Figure 4C).

### Radiotherapy Stratification Analysis

**Figure 5:**
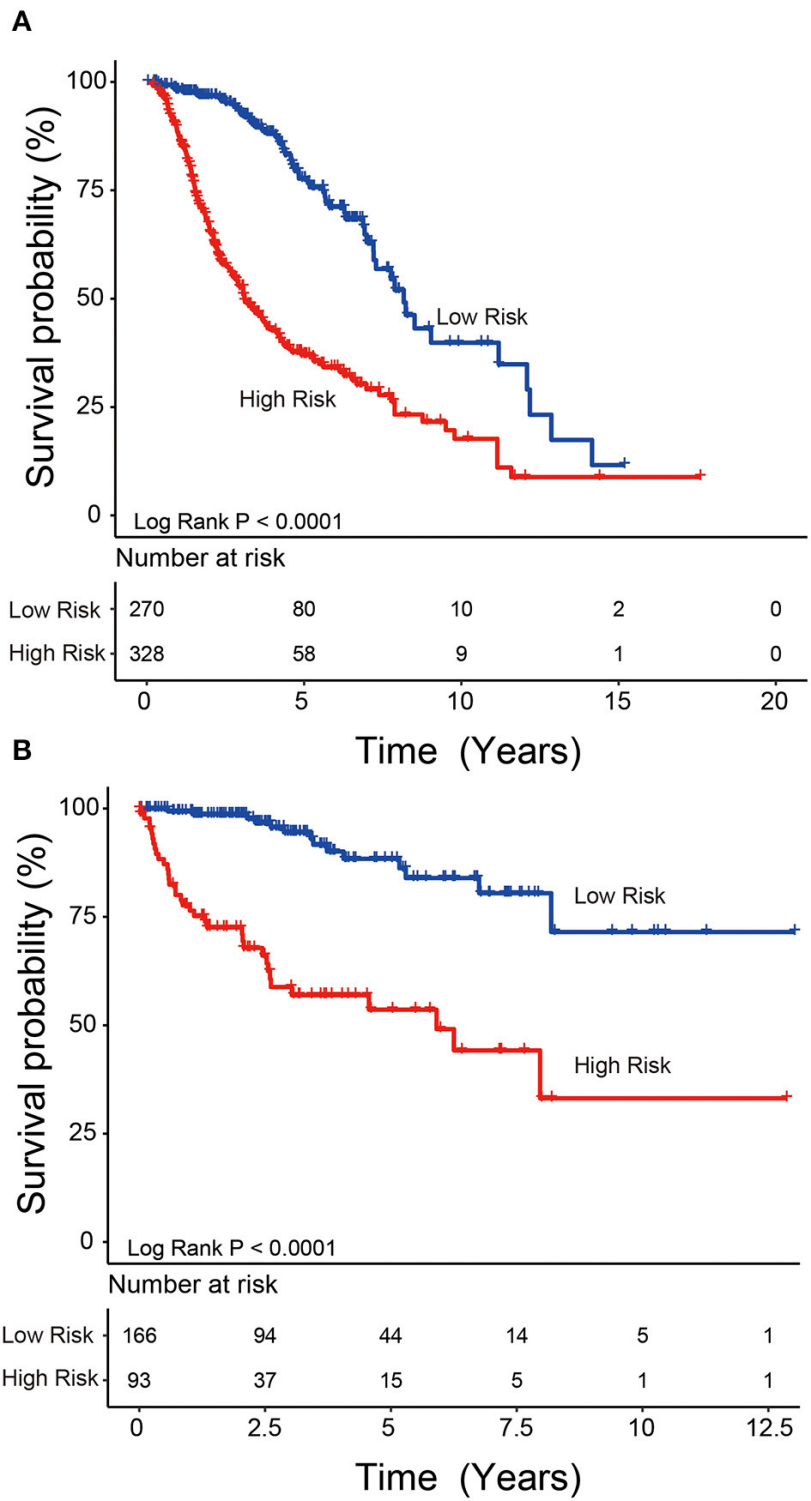
Radiotherapy stratification analysis. The five-PCG signature could further divide patients with radiotherapy **(A)** or patients without radiotherapy **(B)** into two groups with significantly different survival.

Because radiotherapy is the most commonly used treatment in LGGs, we further explore the clinical value of the signature in LGG patients treated with radiotherapy in TCGA and CGGA. According to the radio-status information of all the 955 LGG patients, we found that 598 received radiotherapy, 259 patients did not, and 98 patients had unknown radiotherapy information. For patients after radiotherapy, the five-PCG signature could further divide patients into low- and high-risk groups with significantly different survival (5- or 10-year survival: 77.70/39.84% vs. 37.10/17.69%, log-rank test *P* < 0.001; Figure 5A). Patients without radiotherapy can also be grouped into different risk groups by the five-PCG signature (5- or 10-year survival: 88.39/71.53% vs. 53.59/33.15%, log-rank test *P* < 0.001; Figure 5B).

### Function Prediction for the Five Selected PCGs

**Figure 6:**
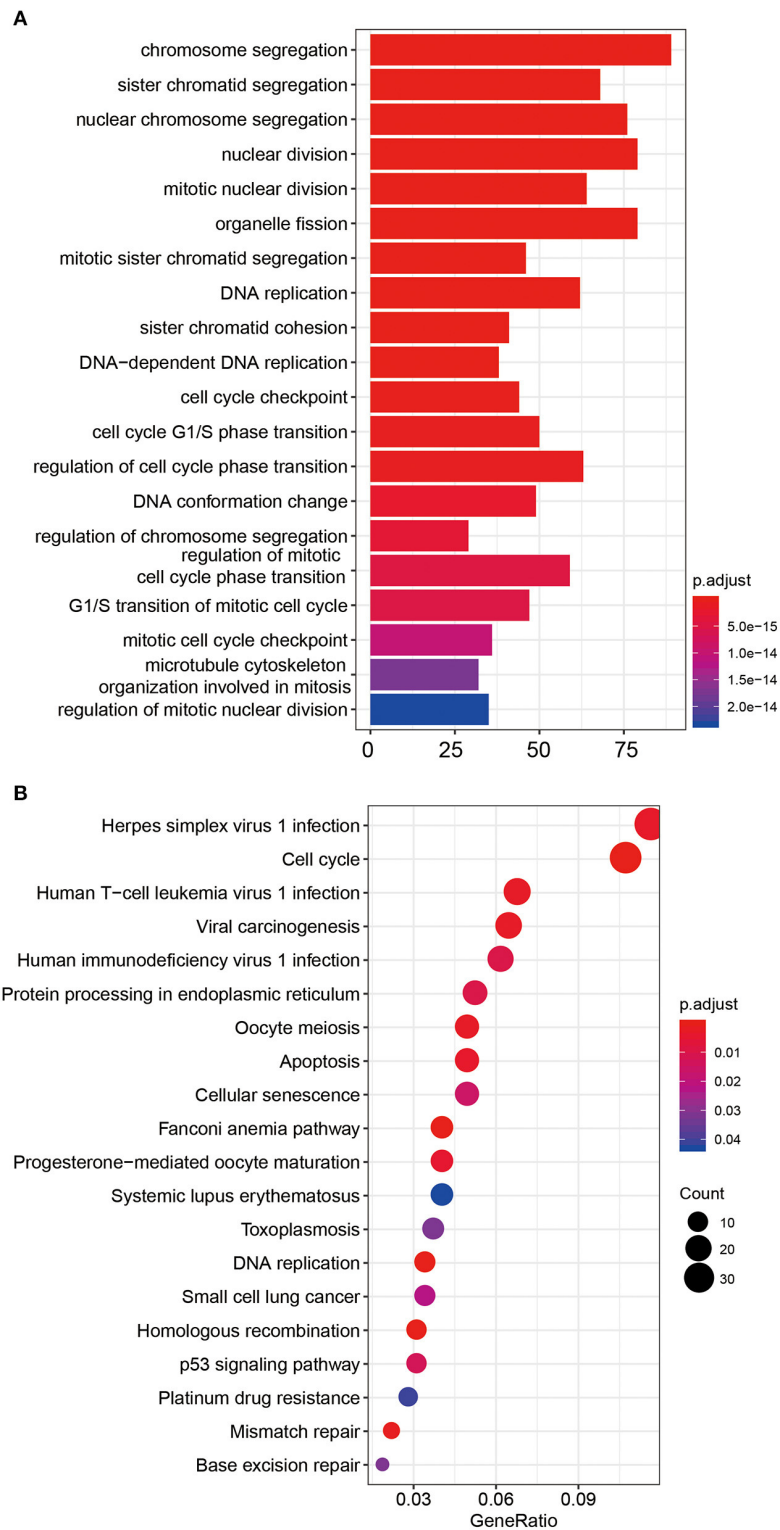
GO **(A)** and KEGG **(B)** functional enrichment analysis of the five PCGs in the signature.

To explore the role and function of the five selected PCGs screened in this study, we obtained a total of 741 co-expressing PCGs (Pearson coefficient >0.5/ <−0.5, *P* < 0.05) using the Pearson test in the TCGA and CGGA datasets, respectively, and then performed KEGG and GO analysis. The co-expressing genes of the five PCGs were significantly enriched in 425 Go terms and 21 KEGG pathways (*P* < 0.05), such as cell cycle, DNA replication, and p53 signaling pathway, indicating the specific pathway or mechanism in which the prognostic PCGs might play a key role (top 20 shown; Figure 6).

## Discussion

The heterogeneity among patients is a major contributing factor in the adverse clinical outcome of gliomas (12). Consequently, the latest edition (2016 edition) of the WHO glioma classification incorporates molecular features into the classification criteria, thereby improving the homogeneity of clinical outcomes in patients with the same subtype (1). However, as one of histological subtypes of glioma, LGG has substantial variation in patient survival and lacks effective prognostic markers. In the current study, we analyzed the survival and gene expression information of 955 patients with LGG and found that the five-PCG signature could be a good prognostic molecular marker. In addition to predicting prognosis of LGG patients, the five-PCG signature has also been found to have a role in guiding radiotherapy.

Tumor heterogeneity and therapeutic advancements have prompted clinicians to make individualized prognosis and treatment choices for cancer patients, thereby achieving precision medicine. Gene biomarkers have always been at the forefront of the development of personalized medicine, especially in the field of cancer. Gene signature-based RNA expression obtained by analyzing gene profiling has been shown to predict the tumor behavior and to distinguish patients with specific tumor grades and/or prognosis (13, 14) in various types of cancer, such as esophageal squamous cell carcinoma, hepatocellular carcinoma, bladder carcinoma, breast cancer, and glioblastoma. In the current study, we aimed to analyze the gene expression profile and develop an effective gene signature for accurate prognosis prediction of LGG patients. After a detailed analysis of gene expression profiles of 955 patients with LGG from the TCGA training set and CGGA validation set, a five-PCG-based prognostic risk model and the five-PCG signature that could distinguish LGG patients with high risk of poor prognosis from patients with low risk were developed. The five-PCG signature has the following two advantages in prognosis prediction: First, it is an independent factor and does not depend on known prognostic factors such as IDH mutation and tumor grade II/III; second, it has excellent prediction performance for its AUC value was higher than IDH mutation and tumor grade.

Notably, the five-PCG signature was found to be a predictive marker for radiotherapy in LGG patients. More specifically, the marker can identify who can benefit from radiotherapy or who is suitable for radiotherapy. As a result, LGG patients have more scientific guidance on whether to accept radiotherapy, and clinicians can also have more standardized guidelines for radiotherapy to facilitate their implementation. This finding shows that the five-PCG signature not only makes the prognosis assessment of patients more precise but also can play the role of individualized treatment. In addition, we noted that the five PCGs in the signature had positive risk factors, meaning they were all prognostic risk factors. By searching the existing literature, we found that the important role of these genes in prognosis prediction had been reported in a variety of tumors. ATP-binding cassette subfamily C member 3 (ABCC3), also named multidrug resistance-associated protein 3 (MRP3), is an organic anion transporter and contributes to drug resistance of cancer cells (15). Consistent with the results in this article, the poor prognosis predictive role of ABCC3 has been reported not only in acute myeloid leukemia (16), gastric cancer (17), pancreatic cancer (18), and lung cancer (19) but also in gliomas (20). In addition to being found as a prognostic marker for gliomas in this article, structural maintenance of chromosomes 4 (SMC4) has also been found to be a survival marker for colorectal cancer (21), breast cancer (22), and prostate cancer (23). Epithelial membrane protein 3 (EMP3) is considered to be a tumor suppressor, but this article found that this gene is a prognostic risk gene for LGG. Similar to our results, Wang et al. (24) also found that EMP3 was associated with the worse prognosis of LGG patients and Guo et al. (25) discovered that EMP3 was also a risk gene in the process of developing a prognostic four-gene panel for glioblastoma patients. WEE1 G2 checkpoint kinase (WEE1) is reported to be an oncogenic nuclear kinase and a regulator of the G2 checkpoint. Expression of WEE1 has been found to be associated with poor prognosis in a variety of tumor types including gliomas (26). Two other gene signatures constructed to predict the prognosis of LGG are also consistent with the results of this article and found that WEE1 is a prognostic risk factor (27, 28). H2B-clustered histone 12 (H2BC12 or HIST1H2BK) is a replication-dependent histone and belongs to the histone H2B family. The prognostic role of HIST1H2BK was identified in ovarian cancer (29), breast cancer (30), and pancreatic ductal adenocarcinoma (31). Although we had some findings on the function of the five prognostic genes by KEGG and GO analysis, further functional exploration of these genes is needed.

## Conclusion

Our study developed a prognostic five-PCG signature for LGG patients that can predict individual clinical outcome with high accuracy. Surprisingly, the five-gene signature can also predict radiotherapy response, which makes the biomarker have a broad clinical value.

## Data Availability Statement

Publicly available datasets were analyzed in this study. This data can be found here: TCGA: https://xenabrowser.net/datapages/; CGGA: http://www.cgga.org.cn/download.jsp.

## Author Contributions

QZ and WL: data collection, data analysis, interpretation, and drafting. X-JL, R-AX, LC, and ZS: study design, study supervision, and final approval of the manuscript. S-BL and F-CX: technical support and critical revision of the manuscript. All the authors read and approved the final manuscript.

## Conflict of Interest

The authors declare that the research was conducted in the absence of any commercial or financial relationships that could be construed as a potential conflict of interest.

## Abbreviations

TCGA: The Cancer Genome Atlas
CGGA: the Chinese Glioma Genome Atlas
ROC: receiver operating characteristic
Kaplan–Meier: KM
AUC: area under the ROC curve
GO: Gene ontology
KEGG: Kyoto Encyclopedia of Genes and Genomes
CI: confidence interval
OS: overall survival.
