# Supplementary figures and images for: Development of a Prognostic Five-Gene Signature for Diffuse Lower-Grade Glioma Patients
Source: Front Neurol. 2021 Jul 6;12:633390. doi: 10.3389/fneur.2021.633390 (PMC8291287; doi:10.3389/fneur.2021.633390)

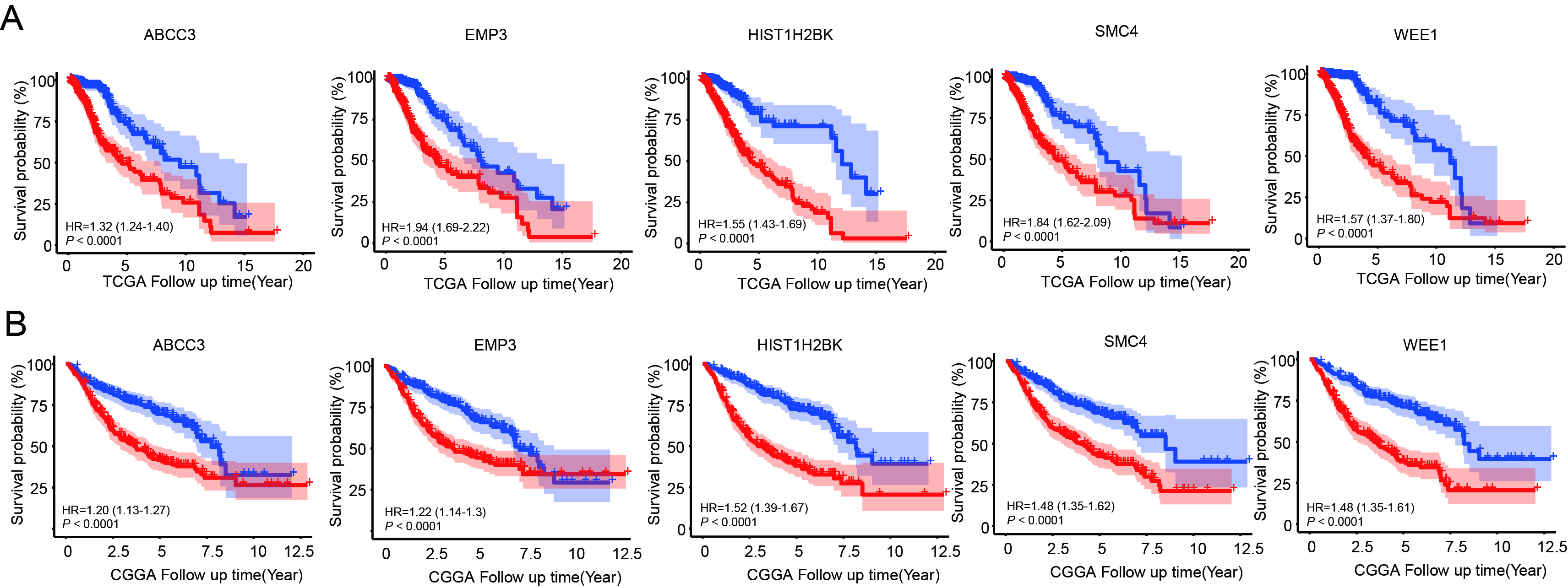

Supplement: Supplementary file 4 [file Image_1.TIF]

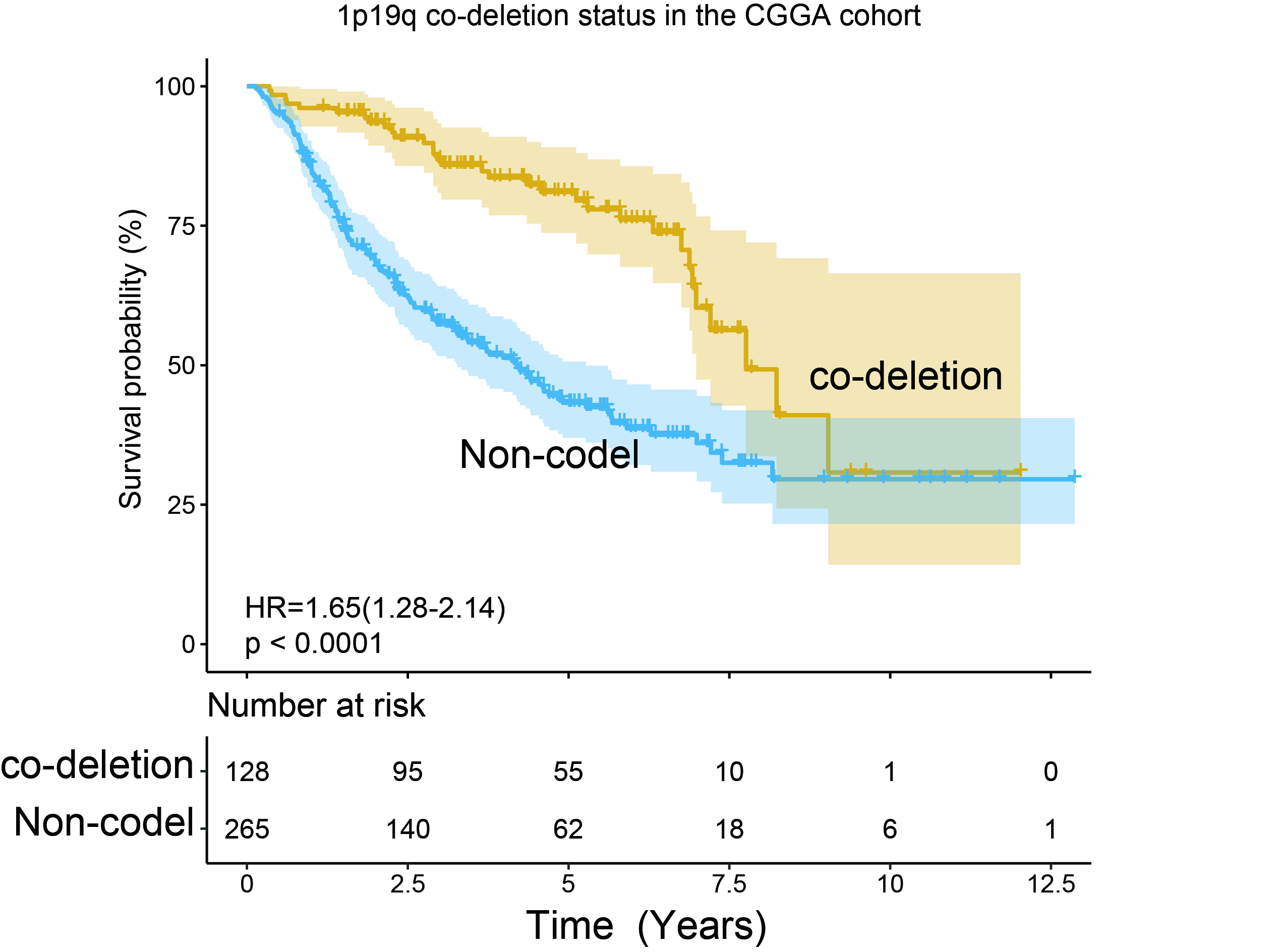

Supplement: Supplementary file 5 [file Image_2.TIF]
